# Clinical predictors and significance of adherent periadrenal fat in laparoscopic adrenalectomy

**DOI:** 10.1186/s12894-023-01348-w

**Published:** 2023-11-01

**Authors:** Erkan Olcucuoglu, Samet Senel, Emre Uzun, Kazim Ceviz, Huseyin Gultekin, Hasan Batuhan Arabaci, Antonios Koudonas, Cevdet Aydin

**Affiliations:** 1 Department of Urology, Ankara City Hospital, Üniversiteler, Bilkent Blv. No:1, Ankara, Çankaya, 06800 Turkey; 2 School of Medicine, First Department of Urology, Aristotle University of Thessaloniki, Thessaloniki, Greece; 3 Department of Endocrinology, Ankara City Hospital, Ankara, Turkey

**Keywords:** Adherence, Laparoscopic adrenalectomy, Periadrenal fat

## Abstract

**Background:**

Adrenalectomy requires the anatomic preparation of the adrenal gland in the fat-rich retroperitoneal space. In the literature, it was shown that the retroperitoneal fat area affects surgical outcomes in laparoscopic adrenalectomy (LA). Besides the quantity of retroperitoneal fat, its qualitative properties play hypothetically a significant role in the safety profile and perioperative parameters of LA. In this study, we aimed to evaluate the factors associated with adherent periadrenal fat.

**Methods:**

The prospectively obtained demographic, preoperative, intraoperative, and postoperative data of 44 patients who underwent laparoscopic adrenalectomy in our clinic were analyzed retrospectively. The patients were divided into two groups as adherent periadrenal fat (APAF) and non-APAF group. Periadrenal fat tissue was defined as adherent or non-adherent by the attending surgeon according to the difficulty in dissection of the adrenal gland from the surrounding fat tissue during the operation.

**Results:**

The rate of female gender and presence of diabetes mellitus (DM) was higher in the APAF group (respectively, p = 0.038 and p = 0.001). A ROC curve analysis showed that the cut-off point was − 97 HU for APAF. On multivariable analysis using a stepwise regression model, we identified the presence of DM (OR = 5.073; 95% Cl = 2.192–12.387; p = 0.006) and ARFD > -97 HU (OR = 3.727; 95% Cl = 1.898–11.454; p = 0.008) as an independent predictor of APAF.

**Conclusion:**

APAF seems to affect the perioperative outcomes of LA in terms of operation duration but not perioperative complications.

## Background

Adrenalectomy was described for the first time in 1889 by John Knowsley-Thornton and is performed in the context of a variety of clinical conditions/ diagnoses, such as functional adrenal tumors, adrenal masses suspicious for malignancy, pituitary-dependent glucocorticoid overproduction by both adrenals (Cushing’s disease) [1]. In 1992, the laparoscopic approach was presented as a viable option by Michael Gagner for performing adrenalectomy, and in the following years, the effectiveness of this approach in eradicating the respective endocrinal disorders was demonstrated [2].

The further evolvement of the laparoscopic approach took place in the period to the present, and nowadays, laparoscopic adrenalectomy (LA) is performed transperitoneally, retroperitoneally, hand-assisted, or robotic-assisted. Regarding the comparison between transperitoneal and retroperitoneal LA, indications, but not robust evidence, exist about reduced late morbidity after retroperitoneal LA [3]. Furthermore, performing the procedure robotically was shown to produce comparable perioperative and postoperative results, and was equally safe with the laparoscopic approach [4].

Since the resection of adrenal masses requires the anatomic preparation of the adrenal gland in the fat-rich retroperitoneal space, several investigators focused on the possible association between the quantitative, qualitative properties of retroperitoneal fat and the complexity of the procedure of LA, or the rate of perioperative/ postoperative complications [5–7].

Besides the quantity of retroperitoneal fat, its qualitative properties play hypothetically a significant role in the safety profile and perioperative parameters of LA. Particularly, the presence of dense fat, which hinders the preparation of the adrenal gland in the retroperitoneal space, may elongate the operation duration and render the procedure more complication-prone. To examine the above hypothesis, we recorded the perioperative/ postoperative data of patients, who underwent LA in our clinic. Fat density was evaluated through radiological imaging, and the state of complicated anatomic preparation was recognized intraoperatively by the surgeon performing the procedure.

## Methods

Our study was prepared following the principles of the Declaration of Helsinki and was approved by the Ankara City Hospital Ethics Committee (Ethics Committee approval number: E2-23-3588).

Forty-four patients who underwent laparoscopic adrenalectomy in our clinic between 07.2019 and 01.2023 were included in the study. The prospectively obtained data of the patients were analyzed retrospectively. Demographic (age, gender, BMI), preoperative (diagnosis, hormonal evaluation, tumor size, laterality, American Society of Anesthesiologists [ASA]score, presence of diabetes mellitus [DM], presence of hypertension [HT], adrenal-renal fat density [ARFD]), intraoperative (operation duration, amount of bleeding, complications, presence of adherent periadrenal fat [APAF]) and postoperative (complications, hospitalization, histological diagnosis) data of all patients were recorded.

All patients were evaluated endocrinologically with patient history, physical examination, laboratory tests (serum cortisol and plasma adrenocorticotropic hormone levels, overnight 1 mg dexamethasone suppression test and /or low-dose two-day dexamethasone suppression test and /or 24-hour urinary free cortisol level, serum aldosterone, serum renin activity, plasma and/or 24-hour urinary catecholamine metabolites) for functionality and radiological methods (computed tomography and/or magnetic resonance imaging).

**Fig. 1:**
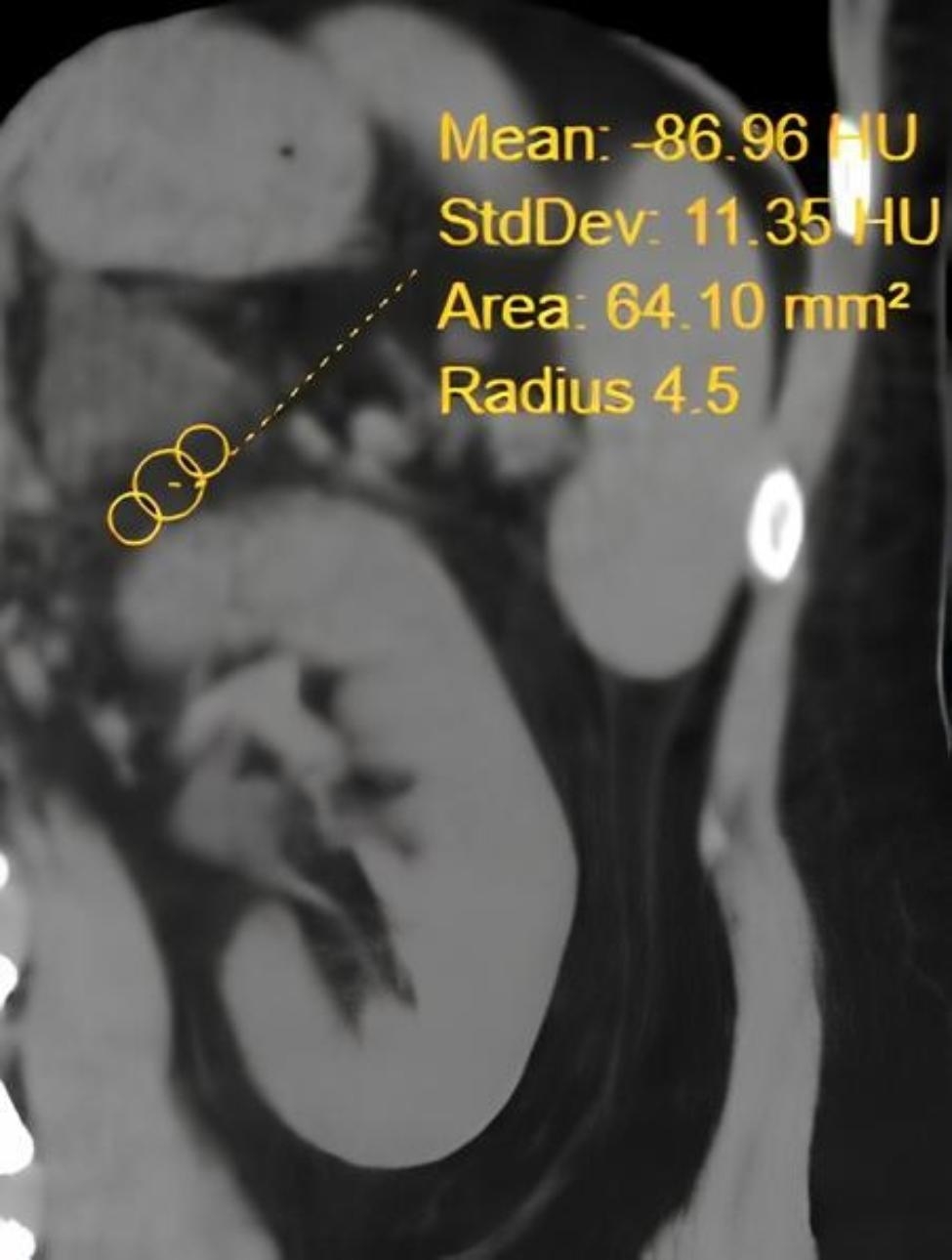
Measurement of adrenal-renal fat density

ARFD was measured on preoperative noncontrast-enhanced computed tomography. Region of Interest (ROI) was localized to fat tissue between the adrenal gland and upper pole of the kidney on the level of the superior border of the adrenal gland for the measurement **(**Fig. 1**)**.

Periadrenal fat tissue was defined as adherent or non-adherent by the attending surgeon according to the difficulty in dissection of the adrenal gland from the surrounding fat tissue during the operation. The patients was divided into two groups as APAF and non-APAF group.

### Surgical technique

All operations were performed transperitoneally by two surgeons (29 cases by S.S. and 14 cases by E.O.). The patients were placed in the semi-lateral decubitus position using table flexion. A 10 cm cloth tape was used to fix the patient at the level of the chest, hip and ipsilateral arm. Initial access was achieved through the camera port using a 12 mm trocar. A 30° endoscope and two additional 10 mm trocars were inserted. The fourth trocar was used in some right side cases if necessary. Intraabdominal pressure was set to 12 mm Hg. The hepatic or splenic flexure of the colon was mobilized medially. Retroperitoneal space was reached. Firstly, the adrenal hilum was identified at the level of renal vein or vena cava and ligated. Then, the adrenal gland was dissected from the surrounding tissues at the kidney upper pole border by opening Gerota fascia. The same procedure was performed at the border of the spleen/liver. The adrenal gland was mobilized and taken out with an endoscopic bag. The urethral foley was removed on the 1st postoperative day.

### Statistical analysis

Data coding and statistical analyses were performed on the computer using the SPSS 22 software package program (IBM SPSS Statistics, IBM Corporation, Chicago, IL). The conformity of the variables to the normal distribution was examined using the Shapiro-Wilk tests. Normally distributed variables were expressed as mean ± standard deviation, and non-normally distributed variables were expressed as median (minimum-maximum) values. The Mann-Whitney U test of non-categorical parameters between groups was used. Chi-square or Fisher’s exact tests were used for categorical variables. Parameters differing between APAF and non-APAF groups were determined. The predictive property of ARFD for APAF in patients undergoing laparoscopic adrenalectomy was analyzed with the receiver operating characteristic (ROC) curve at 95% confidence interval. Whether these parameters were independent risk factors for the presence of APAF was evaluated by multivariate analysis using the Backward LR method. Cases with a p value below 0.05 were considered statistically significant.

## Results

The mean age of 44 patients was 50.8 ± 10.9 years, and the median BMI was 30.2 ± 6.6 kg/m^2^. 28 (63.6%) of the patients were female. Median tumor size was 40.5 (12–84) mm. 14 (31.8%) of the patients were in the APAF group. The rate of female gender and presence of DM was higher in the APAF group (respectively, p = 0.038 and p = 0.001). Median ARFD was statistically significantly higher in the APAF group (-91.5 HU vs. -102 HU, p = 0.001) The demographic, preoperative, intraoperative, and postoperative characteristics of the patients were summarized in Table 1.

**Table 1:** Comparison of demographic, preoperative, intraoperative and postoperative characteristics according to adherent periadrenal fat status of patients who underwent laparoscopic adrenalectomy

Characteristics	Total (n = 44)	APAF group (n = 14, % 31.8)	Non-APAF group (n = 30, % 68.2)	p
**Demographic**				
**Age (year) (Mean ± SD)**	50.8 ± 10.9	49.9 ± 10.3	51.3 ± 11.3	0.711^t^
**Gender (female) n, (%)**	28 (63.6)	12 (85.7)	16 (53.3)	**0.038** ^**c**^
**BMI (kg/m** ^**2**^ **) (Median)(min-max)**	30.2±6.6	32.1±4.6	29.4±7.1	0.158^m^
**Preoperative**				
**Preoperative diagnosis**				
**Pheochromocytoma, n (%)**	14 (31.8)	3 (21.4)	11 (36.7)	0.348^f^
**Cushing’s syndrome, n (%)**	9 (20.5)	5 (35.7)	4 (13.2)	
**Conn’s syndrome, n (%)**	2 (4.5)	0 (0)	2 (6.7)	
**Myelolipoma, n (%)**	4 (9.1)	2 (14.3)	2 (6.7)	
**Suspect of malignancy, n (%)**	15 (34.1)	4 (28.6)	11 (36.7)	
**Hormonal activity**				
**Functional, n (%)**	25 (56.8)	8 (57.1)	17 (56.7)	0.976^c^
**Non-functional, n (%)**	19 (43.2)	6 (42.6)	13 (43.3)	
**Tumor size (mm) (Median)(min-max)**	40.5 (12–84)	37 (29–60)	42 (12–84)	0.412^ m^
**Previous surgery history on surgical side, n (%)**	6 (13.6)	2 (14.3)	4 (13.3)	0.812^c^
**Laterality**				
**Left, n (%)**	19 (43.2)	8 (57.1)	11 (36.7)	0.202^c^
**Right, n (%)**	25 (56.8)	6 (42.9)	19 (63.3)	
**ASA score**				
**1, n (%)**	4 (9.1)	0 (0)	4 (13.3)	0.435^f^
**2, n (%)**	27 (61.4)	10 (71.4)	17 (56.7)	
**3, n (%)**	13 (29.5)	4 (28.6)	9 (30)	
**Presence of HT, n (%)**	21 (47.7)	9 (64.3)	12 (40)	0.133^c^
**Presence of DM, n (%)**	18 (40.9)	11 (78.6)	7 (23.3)	**0.001** ^**c**^
**ARFD (HU) (Median)(min-max)**	-102 (-110 to -65)	-91.5 (-106 to -65)	-102 (-110 to -88)	**0.001** ^** m**^
**Intraoperative**				
**Operation duration (min) (Median)(min-max)**	65 (40–128)	76 (45–128)	60 (40–90)	**< 0.001** ^**t**^
**Amount of bleeding (mL) (Median)(min-max)**	57.5 (20–200)	62.5 (30–140)	50 (20–200)	0.103^ m^
**Intraoperative complications, n (%)**	0 (0)	0 (0)	0 (0)	1^c^
**Postoperative**				
**Postoperative complication, n(%)**	7 (15.9)	2 (14.2)	5 (16.6)	0.624^c^
**Clavien-Dindo classification system**				
**Grade 1**				
**Fever, n**	3	1	2	
**Grade 2**				
**Pulmonary embolism, n**	1	0	1	
**Wound infection, n**	2	1	1	
**Grade 3**				
**Incisional hernia, n**	1	0	1	
**Hospitalization (Median)(min-max)**	2 (1–12)	2 (1–12)	2.5 (2–11)	0.448^ m^
**Histological diagnosis**				
**Adrenocortical adenoma, n**	22	4	18	
**Adrenocortical hyperplasia, n**	3	3	0	
**Pheochromocytoma, n**	9	1	8	
**Myelolipoma, n**	4	2	2	
**Carcinoma metastasis, n**	3	2	1	
**Epithelial cyst, n**	2	1	1	
**Schwannoma, n**	1	1	0	

**APAF**: Adherent periadrenal fat, **BMI**: Body Mass Index, **ASA**: American Society of Anesthesiologists, **HT**: Hypertension **DM**: Diabetes Mellitus, **ARFD**: Adrenal-Renal Fat Density, **HU**: Houndsfield Unit, ^**t**^: Independent Sample T Test, ^**m**^: Mann Whitney U Test, ^**c**^: Chi-kare Test, ^**f**^: Fisher’s Exact Test

Bold p value characters indicates as statistically significant

A ROC curve was produced with a 95% confidence interval for demonstrating the predictive value of ARFD for APAF and the cut-off point was determined as -97 HU (AUC = 0.801, CI: 0.655–0.948; p = 0.001). On multivariable analysis using a stepwise regression model, we identified the presence of DM (OR = 5.073; 95% Cl = 2.192–12.387; p = 0.006) and ARFD > -97 HU (OR = 3.727; 95% Cl = 1.898–11.454; p = 0.008) as independent predictors of APAF **(**Table 2**)**.

**Table 2:** Determination of risk factors for adherent periadrenal fat in patients undergoing laparoscopic adrenalectomy by multivariate logistic regression analysis

Parameters	OR (95% CI)	p
**Gender (female)**	1.643 (0.232–11.618)	0.619
**Precence of DM**	5.073 (2.192–12.387)	**0.006**
**ARFD > -97 HU**	3.727 (1.898–11.454)	**0.008**

**CI**: Confidence Interval, **ARFD**: Adrenal-Renal Fat Density, **DM**: Diabetes Mellitus

Bold p value characters indicates as statistically significant

## Discussion

The current study demonstrates that besides the fat quantity in the retroperitoneum, fat adherence can render the preparation of anatomic planes during LA challenging. Moreover, the state of increased fat adherence seems to be predictable by macroscopic parameters, which are evaluated in the preoperative setting.

The patients participating in the study were bearing a middle-sized adrenal lesion, which was benign in the majority of the cases. Approximately one-third of the cohort was characterized by increased difficulty during the separation of the adrenal gland from the surrounding retroperitoneal fat. This patient subset, named as APAF group, was comparable to the rest patient cohort (non-APAF group) in terms of disease characteristics, perioperative/ postoperative outcomes, except the female ratio, the presence of DM, the ARFD, and the procedure duration, which were significantly different between comparing groups. Theoretically, ARFD represents the most informative parameter for the prediction of the APAF status, and the analysis showed that with the application of an optimum cut–off point, its accuracy in discriminating between APAF, and non-APAF was high. Additionally, the multivariate analysis demonstrated that ARFD remained a significant predictor of APAF status after adjustment for the effect of gender and DM. Interestingly, DM was also of independent predictive value, which suggests a possible aetiological relationship to the increased fat adherence of the APAF patients. APAF status rendered LA more challenging, which was reflected in procedure duration, but not in perioperative outcomes (complications, blood loss), or hospitalization duration. This fact suggests that increased fat adherence can be counterbalanced by a more meticulous and time-consuming anatomic preparation so that the safety of the procedure remains unaffected.

After a thorough literature search, we found several scientific reports, which demonstrate the effect of increased retroperitoneal fat adherence also in renal surgery. According to a systematic review from Khene et al. adherent perinephric fat was associated with a longer procedure duration and higher blood loss during partial nephrectomy, while postoperative complications, margin status, and hospitalization duration were not affected. The same effect was found also in renal surgery for benign conditions, such as the living-donor nephrectomy [8]. According to the report of Narita et al., the increased complexity in the anatomic preparation of the kidney in approximately 50% of the donor group was independently associated with the finding of stranding in the preoperative cross-sectional imaging. Interestingly, in the subgroup of increased fat adherence, a significantly higher concentration of inflammation-related cytocines was measured in the perinephric adipose tissue [9].

Regarding the reports on the prediction and effect of adherent fat in LA, investigators used the Mayo Adhesive Probability (MAP) score, which was designed for the radiological characterization of perinephric fat in patients planned for partial nephrectomy, to predict the presence of adverse conditions during anatomic preparation of the adrenal. In 2022, Kira et al. examined the factors contributing to the elongation of the time duration of LA and concluded that the MAP score was the only independent parameter with the above effect [10]. In the same year, Chen et al. studied the perioperative outcomes of retroperitoneal LA and recognized MAP as the only factor affecting independently all of the evaluated outcomes (operation time, blood loss, hemoglobin drop) [11]. MAP score was also used by the investigators of a third study to evaluate the factors affecting operation time in retroperitoneal LA [12]. MAP score and tumor size were independent parameters of extended procedure duration. Regarding the association of DM with adherent fat, we found similar evidence only in the literature of renal surgery, where DM was one of the independent predictors of encountering adherent fat during partial nephrectomy [13]. In another study, DI Maida et al. investigated the clinical predictors and significance of adherent perinephric fat density at the time of partial nephrectomy. In this study, metabolic syndrome was confirmed as an independent predictor of adherent perinephric fat and adherent perinephric fat did not impact on intra- or perioperative outcomes. These results are consistent with our results. Differently, APAF was associated with longer operation duration in our study [14].

The results of the current study are applicable in the delineation of the challenging cases of LA, where increased expertise is needed to maintain perioperative events and postoperative morbidity at the lowest possible level. More studies are needed to consolidate the above results and to further optimize the procedure of LA.

## Conclusion

Adherent fat, which is recognized as an adverse parameter complicating the anatomic preparation of the kidney in renal surgery, seems to affect the perioperative outcomes of LA, at least in terms of operation duration. ARFD comprises an adequately precise predictor of adherent fat, which can be evaluated in the preoperative setting and seems to correlate independently with the APAF status.

## Data Availability

The datasets generated and/or analyzed during the current study are available in Figshare Repository at https://figshare.com/s/d77b9b53490959599bd0.

## List of abbreviations

LA: Laparoscopic adrenalectomy
APAF: Adherent periadrenal fat
DM: Diabetes mellitus
BMI: Body mass index
ASA: American Society of Anesthesiologists
HT: Hypertension
ARFD: Adrenal-renal fat density
APAF: Adherent periadrenal fat
ROI: Region of Interest
MAP: Mayo Adhesive Probability
